# Impact of sarcopenia and obesity on skeletal muscle size, gene expression, and mitochondrial function

**DOI:** 10.1007/s11357-025-01726-2

**Published:** 2025-06-12

**Authors:** Hector G. Paez, Christopher R. Pitzer, Jessica L. Halle, Peter J. Ferrandi, James A. Carson, Junaith S. Mohamed, Stephen E. Alway

**Affiliations:** 1 Department of Physiology, College of Medicine, University of Tennessee Health Science Center, Memphis, TN 38163 USA; 2 Integrated Biomedical Sciences Graduate Program, College of Graduate Health Sciences, University of Tennessee Health Science Center, Memphis, TN 38163 USA; 3 Laboratory of Muscle Biology and Sarcopenia, Division of Regenerative and Rehabilitation Sciences and Department of Physical Therapy, College of Health Professions, University of Tennessee Health Science Center, Memphis, TN 38163 USA; 4 Integrative Muscle Biology Laboratory, Division of Regenerative and Rehabilitation Sciences, College of Health Professions, University of Tennessee Health Science Center, Memphis, TN 38163 USA; 5 Laboratory of Muscle and Nerve, Department of Diagnostic and Health Sciences, College of Health Professions, University of Tennessee Health Science Center, Memphis, TN 38163 USA; 6 Center for Muscle, Metabolism and Neuropathology, Division of Rehabilitation Sciences, College of Health Professions, University of Tennessee Health Science Center, Memphis, TN 38163 USA; 7 Current address: Department of Comparative Biosciences, College of Veterinary Medicine, University of Wisconsin Madison, Madison, WI 53706 USA; 8 Current Address: Center for Exercise Medicine Research, Fralin Biomedical Research Institute at VTC, Roanoke, VA 24016 USA; 9 Current address: Oklahoma Medical Research Foundation, Oklahoma City, OK 73104 USA; 10 Current Address: Integrative Muscle Biology Laboratory, Department of Kinesiology & Sports Management Department, Huffines Institute for Sports Medicine & Human Performance, Texas A&M University College Station, College Station, TX 77843 USA

**Keywords:** Aging, Obesity, Skeletal muscle, Sarcopenic obesity, Mitochondria, Metabolism

## Abstract

Skeletal muscle is a primary tissue of dysfunction during both aging and obesity. Recently, the coincidence of obesity and aging has gained attention due to the intersection of the obesity epidemic with an aging demographic. Both aging and obesity are associated with marked defects in skeletal muscle metabolic health. Despite these findings, we have a poor understanding of how obesity and aging may interact to impact skeletal muscle mass and metabolic health. Therefore, we investigated the impact of high-fat diet (HFD)-induced obesity on skeletal muscle mass, mitochondrial function, transcriptomics, and whole-body metabolism in young and aged mice. We observed main effects of diet and age on several measures of whole-body metabolic function (VO_2_, VCO_2_, and RER). Complex I-driven mitochondrial proton leak was significantly elevated by HFD-induced obesity across both age groups; however, a main effect of aging for reduced complex I leak was detected in the soleus muscle. Interestingly, aged animals fed a HFD did not exhibit lower muscle mass than chow-fed young animals, but did present with stark increases in muscle triglyceride content and a unique transcriptional response to HFD. HFD-induced obesity impacted the muscle transcriptome differently in the muscles of young and aged mice, indicating that obesity can change altered gene expression with age. Our findings suggest that the presence of obesity can both compound and counteract age-associated changes to muscle mass, gene expression, and mitochondrial function.

**Supplementary Information:**

The online version contains supplementary material available at 10.1007/s11357-025-01726-2.

## Introduction

With advancing age, the preservation of skeletal muscle mass and quality is crucial to maintain overall metabolic health, independence, and mobility. The loss of skeletal muscle mass and function with aging (i.e., sarcopenia) can contribute to the development of disability and thus represents a significant fiscal and societal burden [1, 2]. Significant medical advances have contributed to an extension of human lifespan; the World Health Organization projects that one in six people will be over the age of 65 by the year 2030 [3]. Despite an increase in human lifespan, human “healthspan,” the length of time a person is free from disability and disease, has suffered as the coincidence of aging-associated and chronic metabolic disease has risen. Sarcopenic obesity, which is the combination of high adiposity and low muscle mass, is an emerging public health concern and highlights the convergence of a global obesity epidemic and the demographic shift towards an aging population. Aging is associated with motor unit loss [4], altered proteostasis [5], mitochondrial dysfunction [6, 7], systemic inflammation [8], and inflammasome activation in skeletal muscle [9]. Currently, nearly a third of US adults are overweight [10]. Furthermore, obesity has been shown to impair insulin sensitivity [11] and, like age-associated sarcopenia, is linked to skeletal muscle mitochondrial abnormalities [12–14]. Sarcopenic obesity is associated with a greater risk of type 2 diabetes [15], cardiovascular disease [16], and hypertension [17], as well as all-cause mortality [18]. Despite these findings, we have a limited understanding of whether aging exacerbates signaling events associated with obesity and vice versa. How obesity impacts the aging phenotype is an important gap in our knowledge, as several preclinical studies compare young lean mice to aged mice that are significantly heavier [19–23] when attempting to determine the effects of aging on skeletal muscle. Therefore, we aimed to investigate the impact of high-fat diet (HFD)-induced obesity on skeletal muscle mitochondrial function, transcriptomics, and whole-body metabolism in young and aged mice. Furthermore, we examined if HFD-induced obesity and aging result in similar or distinct changes in skeletal muscle mass. In the current study, we report that mitochondrial respiratory leak is tightly coupled with body weight and muscle triglyceride content regardless of age and that while HFD-induced obesity results in similar gains in fat mass and body weight between young and aged animals, aged obese animals exhibit a large increase in skeletal muscle triglyceride content when compared to obese young animals. We also observed that aged obese animals expressed several unique differentially regulated genes when compared to young or lean counterparts and that obesity and aging produced divergent effects on hindlimb muscle mass.

## Materials and methods

### Animals

Male and female C57BL/6 J mice (*n* = 4–10 per group) were purchased from The Jackson Laboratory (Bar Harbor, ME) at 12 weeks of age. Mice were aged in-house until they were either 5 months (young) or 23–27 months (aged) of age and then randomized to either young animal chow diet, aged animal chow diet, young animal high-fat diet (HFD), or aged animal HFD groups. The duration of the diet lasted a total of 14 weeks. Mice of 5 months of age were chosen as young controls so that the development of obesity would not impair the developmental growth of skeletal muscles in younger mice. Mice were singly housed during the duration of the diet and kept on a 12:12-h light/dark cycle beginning at 06:00 h and given either standard rodent chow ad libitum (Harlan Teklad Rodent Diet #7912; 5.8% kcal from fat, 3.1 kcal/g) or HFD (Research Diets Rodent Diet D12492; 60% kcal fat, 5.21 kcal/g). Mouse food consumption was weighed once weekly for the first 4 weeks, and the average weekly food intake was then calculated as a daily average. Body weight was measured once weekly for the first 10 weeks of the diet. Prior to euthanasia, mice were fasted for 10 h overnight and euthanized between 08:00 and 10:00. Fasting blood glucose was measured using an Alphatrak 2 veterinary glucometer (Zoetis). Hindlimb muscle mass was calculated as the average of both limbs and plotted as sex-corrected relative muscle mass, wherein each data point is normalized to the sex-specific control (young chow-fed female mice or young chow-fed male mice) group mean. All experiments were approved by the University of Tennessee Health Science Center Animal Care and Use Committee.

### RNA-Seq and bioinformatic analysis

3′ RNA Sequencing was performed on the Aviti Element using Lexogen 3′ RNA Seq library kit at the University of Tennessee Health Science Center Molecular Resource Center (MRC) on RNA isolated from the gastrocnemius muscle (*n* = 6–7/group). Quality control of the raw sequencing reads was conducted using FastQC (version 0.12.1) to assess the quality metrics. Reads were trimmed to remove short reads, poly-As, poly-Gs, and adapter sequences using cutadapt (version 4.8) per the manufacturer’s instructions. Aligned reads were mapped to the mouse genome GRCm39 primary assembly (Gencode release M35) using STAR (version 2.7.11b) with settings per the manufacturer’s instructions. Gene-level count data were generated using STAR (version 2.7.11b) to produce a raw count matrix. GRCm39 annotations (Gencode release M35) were used in generating gene level counts. The gene count data were imported into R (version 4.4.1) and analyzed using the edgeR package (version 4.2.1) [24]. Genes with low counts were filtered out using a threshold of a minimum of 10 reads per gene across all samples, followed by filtering using “filterByExpr” function in edgeR. For the downstream analysis, only protein coding genes and genes coding for microRNA were considered. Differential expression analysis was done using the edgeR package (version 4.2.1) available in R (version 4.4.1) as described elsewhere [25]. Differentially expressed gene (DEG) analysis used the generalized linear model (GLM) framework to model the count data. The glmFit function was applied to fit the model to the filtered gene counts. Subsequently, differential expression analysis was performed using the glmLRT function to identify DEGs. Differential expression results were visualized using several methods: heatmaps of the top 30 DEGs were created using the ggplot2 package (version 3.5.1), employing *z*-score normalization and hierarchical clustering; principal component analysis (PCA) was performed with prcomp() in R programming on log2(CPM + 1) data and visualized using the ggplot2 package to illustrate sample clustering. Volcano plots were created in GraphPad Prism. All visualizations were customized for clarity and saved in high-resolution formats. Analyses were conducted in R (version 4.4.1) using the packages: ggplot2 (version 3.5.1) and edgeR (version 4.2.1). All genes (DEGs and background genes) were then uploaded to the Advaita iPathway platform for gene ontology, Upset plot, upstream regulator predictions, and pathway analysis between groups. LogFC of 0.32 (1.25 FC) and FDR < 0.05 were used as thresholds for DEGs. An FDR < 0.05 was used for reporting gene ontology, upstream regulator predictions, and pathway analysis. Venn diagrams were made with InteractiVenn [26].

### Indirect calorimetry and body composition measurements

Subsets of mice were individually housed and placed in Comprehensive Laboratory Animal Monitoring System (CLAMS; Columbus Instruments) cages 12 weeks after the start of the diet for 48 h total. The animals were maintained in the CLAMS cages at ambient temperature (22 °C) on a 12-h light/dark cycle and had access to their respective diets ad libitum. The first 24-h period in the CLAMS system was used for acclimatization and not analyzed. After acclimatization, oxygen consumption (VO_2_), carbon dioxide production (VCO_2_), and respiratory exchange ratio (RER; VCO2/VO2) were measured. Lean and fat mass were measured using magnetic resonance imaging (EchoMRI 1100 Body Composition Analyzer, Houston, TX). MRI was performed at 12 weeks after the start of the diet, prior to CLAMS experiments. The machine was calibrated before experimental measurements using canola oil, and mice were weighed before Echo MRI analysis.

### High-resolution respirometry of permeabilized muscle fibers

Mitochondrial oxygen flux per skeletal muscle wet weight (pmol·s^−1^·mg^−1^) was determined using an Oxygraph-2 k High-Resolution Respirometer (Oroboros Instruments, Innsbruck, Austria). Measurements were made at 37 °C in the oxygen concentration range of 250–550 µM in 2.0 ml of respiration medium Mir05 (0.5 mM EGTA, 3 mM MgCl2, 60 mM K-lactobionate, 20 mM taurine, 10 mM KH2PO4, 20 mM HEPES, 110 mM sucrose, and 1 g/l BSA, pH adjusted to pH of 7.1 with KOH) as previously described [27]. In brief, two 1–4 mg bundles of fresh soleus muscle tissue were separated and stored on ice in BIOPS preservation buffer (10 mM Ca-EGTA buffer, 0.1 µM free calcium, 20 mM imidazole, 20 mM taurine, 50 mM K-MES, 0.5 mM DTT, 6.56 mM MgCl2, 5.77 mM ATP, 15 mM phosphocreatine, pH 7.1). Each muscle fiber bundle was then mechanically separated using forceps in BIOPS on ice for a maximum of 5 min. After mechanical separation, fiber bundles were permeabilized for 30 min at 4 °C in saponin (50 µg/ml BIOPS) and then washed in Mir05 for 20 min. Following permeabilization, fiber bundles were dried for 30 s on Whatman paper and weighed for a maximum of 30 s. Data were recorded using Datlab 7.3 software (Oroboros Instruments, Innsbruck, AT) at a data recording interval of 2 s. Mitochondrial respiration was assessed using the following sequential titrations of substrates, uncouplers, and inhibitors: (1) 0.1 mM malate and 75 µM palmitoyl-carnitine to measure LEAK respiration supported by electron flow through the electron transferring flavoprotein (ETF; FAO_L_); (2) 5 mM pyruvate, 2 mM malate, and 10 mM glutamate to measure LEAK respiration supported by complex I-linked substrates (CI_L_); (3) 5 mM ADP to determine maximal state 3 respiration supported by fatty acid oxidation and complex I (CI_P_); (4) 10 µM Cytochrome C to assess the quality of the mitochondrial sample and the integrity of the mitochondrial outer membrane (data was not used from chambers if there was a > 15% increase in respiration after cytochrome C addition); (5) 10 mM succinate to determine maximal state 3 respiration with convergent electron input from complex I and II (CI + CII_P_); (6) 0.05 µM stepwise titrations of carbonylcyanide m-chlorophenyl hydrazone to assess electron transfer system capacity (CI + CII_E_); (7) 0.5 µM titration of rotenone to inhibit complex I and fatty acid oxidation and allow for the measurement of complex II supported electron transfer system capacity (CII_ETS_); (8) 2.5 µM of Antimycin A to inhibit complex III and obtain non-mitochondrial residual oxygen consumption for normalization of all other values. Since fatty acid substrates were included at the start of the protocol, all respiratory states prior to the addition of rotenone involved electron transfer through ETF. Respiratory measurements were normalized to tissue wet weight immediately prior to addition into the chamber and muscle tissue used for respirometry was taken from animals euthanized between the hours of 08:00 and 10:00 to ensure similar circadian rhythmicity across animals.

### Tissue collection and histology

At the time of euthanasia, mice were fully anesthetized (3–5% isoflurane) and tissues were rapidly excised, cleared of excessive connective tissue, weighed, and snap frozen in liquid nitrogen and stored at − 80 °C. Hindlimb muscles were collected, including the soleus, plantaris, gastrocnemius, quadriceps, extensor digitorum longus (EDL), and tibialis anterior (TA). The TA muscle was frozen in 2-methylbutane cooled with liquid nitrogen. Tissue cross-sections were cut at an 8 µm thickness at − 20 °C (CM3050S, Leica Biosystems Nussloch, Germany) and mounted on charged glass slides (Thermo Fisher Scientific, Pittsburgh, PA, USA) and kept at − 80 °C prior to immunocytochemistry as previously described [28]. Briefly, muscle sections were air dried for 10 min and rehydrated with PBS for 10 min before being blocked with 2.5% normal goat serum for 1 h at room temperature. Primary antibodies were obtained from the Developmental Studies Hybridoma Bank (Iowa City, IA, USA) dystrophin (MANDYS8(8H11) IgG2b) and incubated overnight at 4 °C. The slides were washed in PBS three times for 5 min each and then incubated in secondary Alexa Fluor antibodies (Thermo Fisher Scientific, Pittsburgh, PA, USA) that consisted of 2 µg/ml in PBS of the Alexa Fluor anti-mouse IgG2b (A21146). The fluorescent images of the muscle cross-sections were captured with a Biotek Lionheart™ FX Automated Microscope (Winooski, VT, USA) and were imaged with emission/excitation at 628/685 nm. Cross-sectional areas and minimum ferret diameters of the fibers in the tibialis anterior muscles were analyzed and measured using the Myovision software [29] from four randomly selected fields at an objective magnification of × 10.

### Skeletal muscle triglyceride content

Quadricep muscles were homogenized NP40S buffer and centrifuged at 500 g for 5 min at 4 °C to pellet cell debris. The cell lysate was assayed in a commercially available colorimetric triglyceride quantification kit according to manufacturer instructions (Cayman Chemicals; #10,010,303) and analyzed against a standard curve. The triglyceride concentration in the lysate was then normalized to protein concentration as measured with a detergent compatible colorimetric protein quantification kit (Biorad, DC™ Protein Assay Kit I #5000111). Data are expressed as muscle triglyceride content relative to young control animals.

### Skeletal muscle mitochondrial DNA quantification

Skeletal muscle (Quadriceps) DNA was isolated using the Invitrogen™ PureLink™ Genomic DNA Mini Kit (Thermo Fisher, #K182001). After purification, mitochondrial DNA (mtDNA) and nuclear DNA (nDNA) were quantified via Sybr Green qPCR using primers designed to amplify the mitochondrial-encoded ND2 gene and the nuclear-encoded PECAM1 gene. The CT value from each gene was utilized to calculate the 2^−ΔΔCT^ and relative quantification of mitochondrial DNA content.

### RNA isolation and gene expression analysis

Total RNA was isolated from whole muscle using TRIzol reagent (Invitrogen, #15,596,026) per manufacturer instructions. After phenol–chloroform extraction, RNA was precipitated using an equal volume of 70% ethanol and purified using the PureLink® RNA Mini Kit (Invitrogen, Carlsbad, CA, USA) before being eluted in nuclease-free water. RNA concentration and purity (260/280 ratio) were measured using a Biotek Take3 plate (Biotek Ref. #TAKE3‐SN). RNA was then treated with DNase I (Invitrogen, #18,068,015) to eliminate any genomic DNA, and cDNA was reverse transcribed from 1 µg of total RNA using the High Capacity cDNA Reverse Transcription kit according to manufacturer instructions (Invitrogen, #4368814). cDNA was then diluted 25-fold and stored at − 80 °C. Quantitative real-time PCR was used to measure gene expression using a Roche LC480 qPCR machine and Roche SYBR green PCR master mix (Roche Ref. #04707516001) as previously described [30]. Melt-curve analysis was performed, and any reaction that produced two melt peaks was omitted from analysis. The 2^−ΔΔCt^ method was used to determine gene expression changes between treatment groups. Housekeeping gene analysis was performed to identify stable housekeeping genes (HPRT, GAPDH, β-actin, RPS20) prior to qPCR analysis.

### Immunoblot

Muscle tissue was homogenized in RIPA buffer (Invitrogen, #89901) supplemented with EDTA and HALT protease/phosphatase inhibitor cocktail according to manufacturer instructions (Invitrogen, #78440). Protein quantification was assessed via the detergent compatible colorimetric protein quantification kit (Biorad, DC™ Protein Assay Kit I #5000111). Western blot analysis of protein abundance and normalization to total protein content was performed as previously described [31]. For the phosphorylation status of proteins, both total target protein and phosphorylated target protein values were normalized to a total protein stain using No-Stain™ Protein Labeling Reagent (Invitrogen, #A44449). The phosphorylated target protein: total target protein ratio was then calculated and plotted relative to control young chow values. When possible, total target protein values were assessed on the same membrane that phosphorylated target protein was probed for after membrane stripping using Restore™ PLUS Western Blot Stripping Buffer (Thermo Fisher, # 46430). However, when residual signal was detected after membrane stripping, a second western blot was performed to quantify total target protein abundance and normalized to total protein before calculating the ratio of phosphorylated: total target protein.

### Statistical analysis

One-way ANOVA was used to compare groups in measurements of starting bodyweight, fiber cross-sectional area (CSA), fiber distribution, and fiber feret diameter. Two-way repeated measures ANOVA was utilized to analyze repeated measures data. Two-way ANOVA with age and diet as independent variables was used to test for main effects in all other measures. Tukey’s post hoc was utilized to determine significant group differences at *p* < 0.05. Data were tested for normality using the Shapiro–Wilk test. When the assumption of normality was violated, data were analyzed using the nonparametric Kruskal–Wallis test with Dunn’s post hoc test or the Brown-Forsythe and Welch ANOVA with Dunnett’s post hoc test when the variance between groups was unequal. Linear relationships between variables were determined using the Pearson *r* correlation coefficient. Results are reported as mean $\pm$ SEM. Statistical analyses were performed using GraphPad Prism. Groups with different letter annotations are significantly different from each other.

## Results

### Impact of obesity on body composition and feeding in young and aged mice

**Fig. 1:**
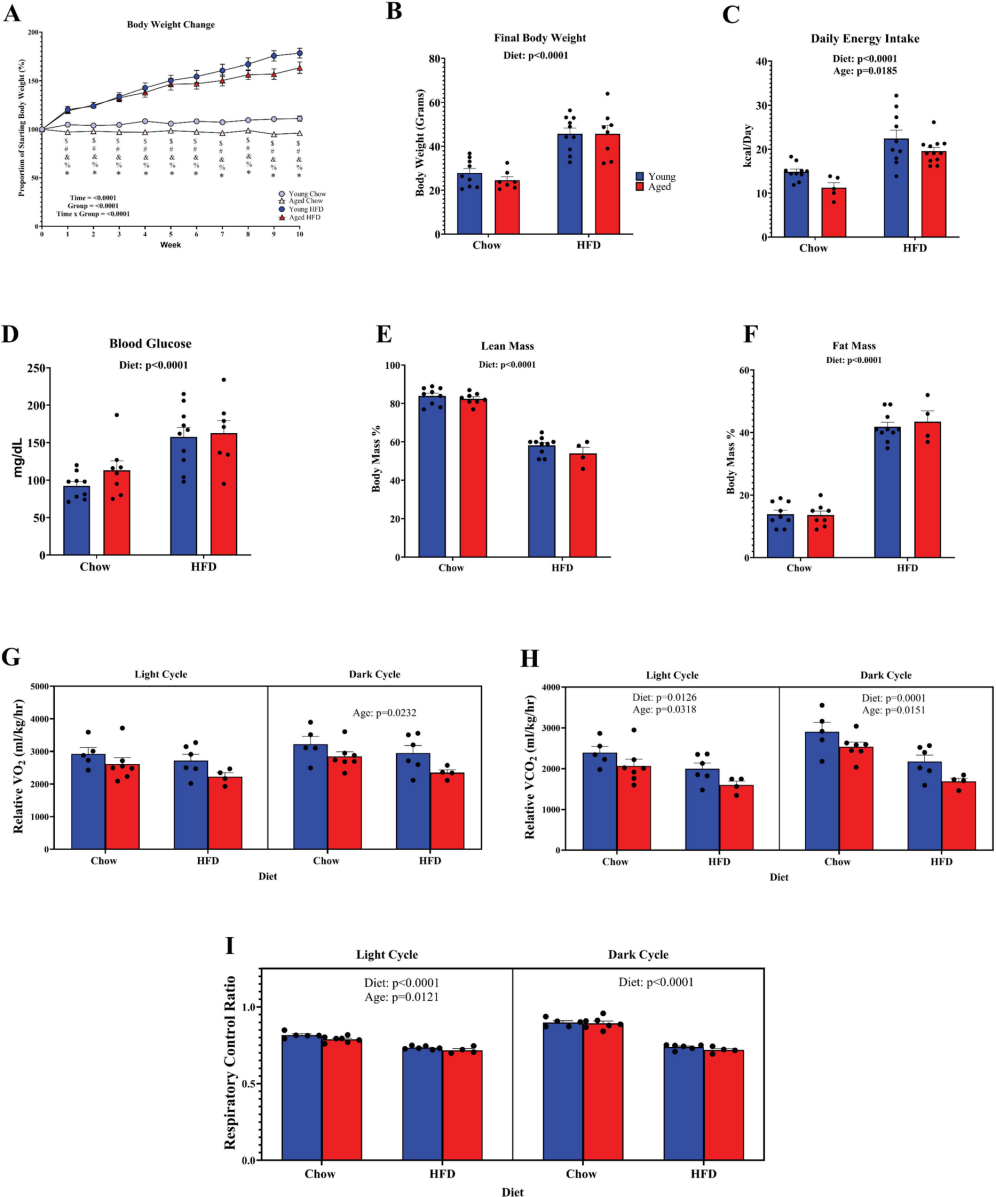
Impact of high-fat diet on body weight, fasting blood glucose, and body composition in young and aged mice. **A** Change in body weight expressed as a percentage of starting body weight over time (*n* = 8–10/group). **B** Final body weight of mice after 14 weeks of either chow or HFD. **C** Average daily caloric intake during these first 4 weeks of the diet. **D** Blood glucose measurement after a 10-h fast. **E** Lean mass and **F** fat mass as a percentage of body weight. Measurements of relative **G** VO_2_, **H** VCO_2_, and **I** RER during the light and dark cycle. **A** *p < 0.05 aged HFD vs. young chow, #*p* < 0.05 aged HFD vs. aged chow, %*p* < 0.05 young chow vs. aged chow, $*p* < 0.05 aged chow vs. young HFD, &*p* < 0.05 young chow vs. young HFD, §*p* < 0.05 young HFD vs aged HFD. Results are reported as mean ± SEM

Young and aged animals tended to increase bodyweight similarly when fed a high-fat diet (Fig. 1A). A main effect of diet was observed for final bodyweight at the end of the study, with young and aged mice weighing approximately 47 g after high-fat diet (HFD) feeding (Fig. 1B). Interestingly, despite the similar weight gain, we observed a main effect of age to reduce caloric intake (*p* = 0.0185) (Fig. 1C). As expected, we detected a main effect of diet (*p* < 0.0001) on fasting blood glucose (Fig. 1D), indicating that the metabolic impairment from the HFD was apparent in both age groups. A main effect of diet was observed for reduced lean mass and enhanced fat mass (Fig. 1E, F), irrespective of age. The effects of obesity and aging on whole-body metabolism were assessed using CLAMS metabolic cages for measures of VO_2_, VCO_2_, and respiratory exchange ratio (RER). We did not observe an effect of age or diet on VO_2_ during the light cycle (Fig. 1G) but did find an effect of age for reduced VO_2_ in the dark cycle, when mice are more active [32, 33]. There were main effects of both age and diet for reduced VCO_2_ during the light and dark cycle (Fig. 1H). We detected a suppression of RER during both the light and dark cycle by diet and an effect of age for reduced RER during the light cycle (Fig. 1I). These data suggest that both obesity and aging influence whole body metabolism in similar ways, but that age does not exacerbate HFD-mediated weight gain or elevated fasting blood glucose.

### Aging and obesity result in divergent effects on muscle mass

**Fig. 2:**
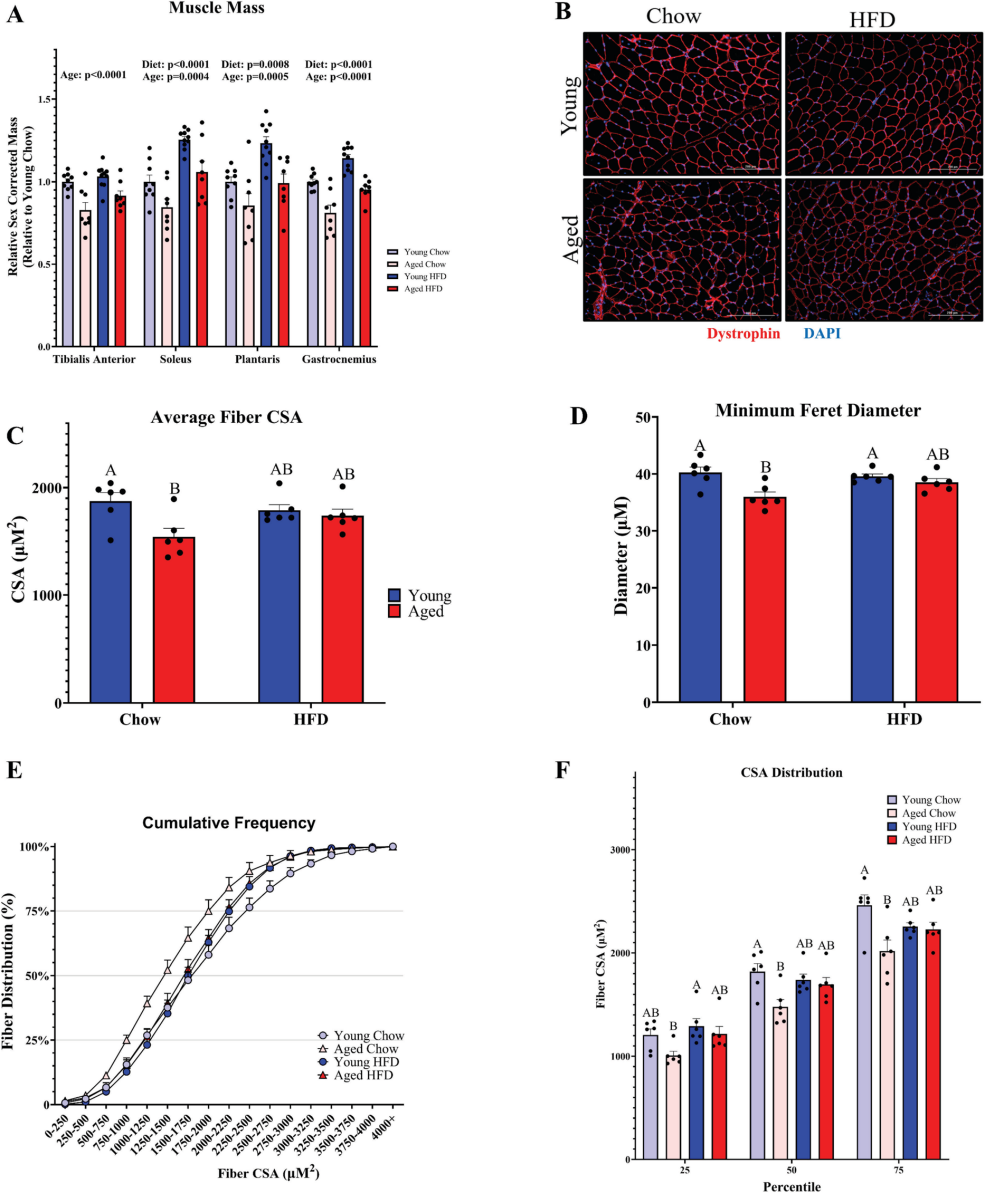
Aging and obesity exhibit divergent effects on muscle mass: **A** Hindlimb muscle mass expressed relative to sex-corrected control values. **B** A representative immunofluorescence section of the TA muscle stained for dystrophin and nuclei (DAPI). Scale bar is 200 µm. **C** Average cross-sectional area (CSA) and **D** average minimum feret diameter of tibialis anterior muscle fibers. **E** Cumulative frequency of fiber distributions and **F** fiber size at the 25 th, 50 th, and 75 th percentiles. Statistical significance was set to *p* < 0.05. Different letters (A, B, C) denote significant differences between groups. Results are reported as mean ± SEM

**Fig. 3:**
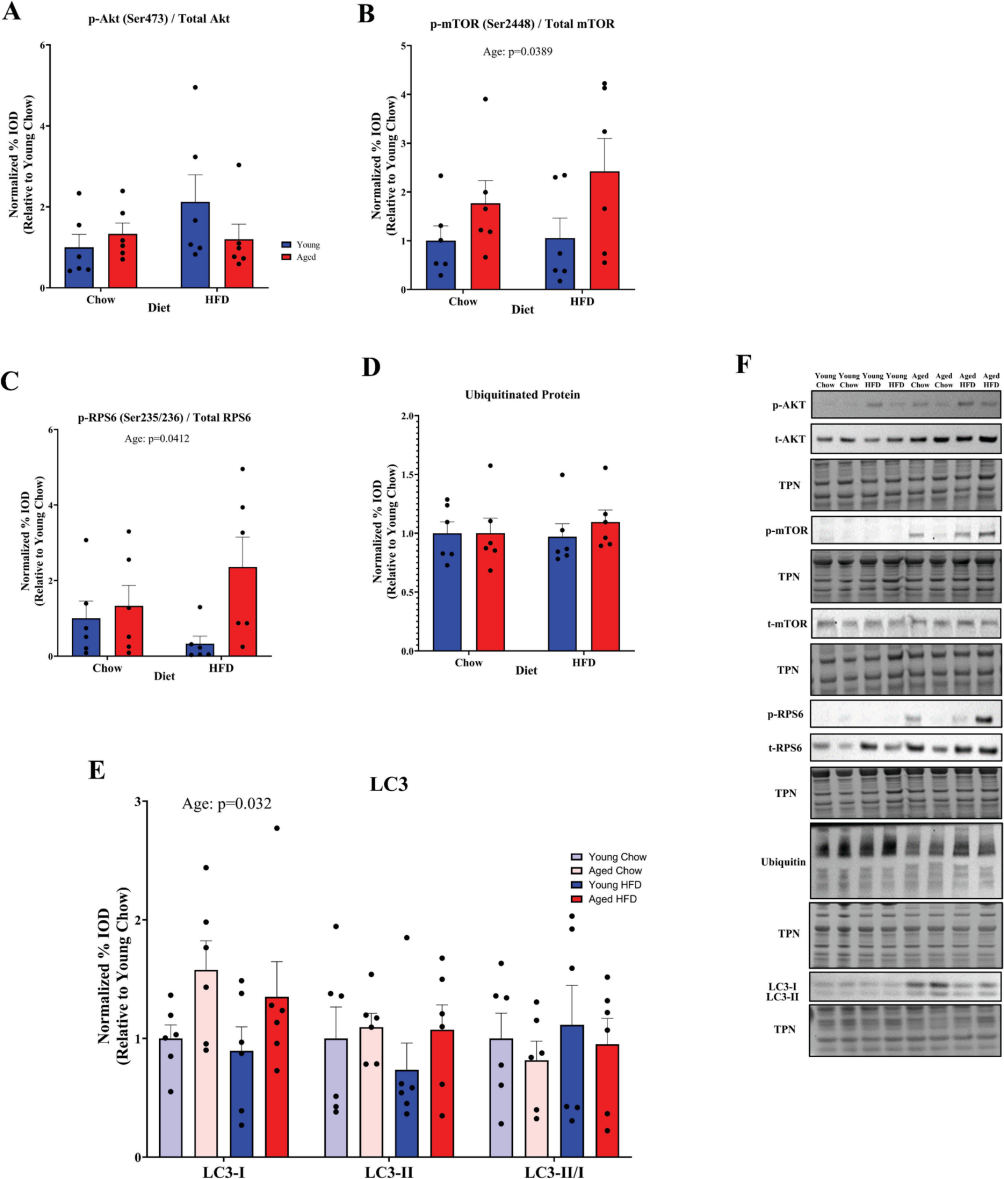
Western blot analysis of the phosphorylation status of proteins involved in skeletal muscle growth in the TA muscle. **A** Phosphorylation of Akt at Ser473, **B** phosphorylation of mTOR at Ser2448, and **C** phosphorylation of RPS6 at Ser235/236. Values shown are phosphorylated values normalized to total target protein abundance and expressed relative to young chow controls. Signaling associated with protein degradation was assessed by measuring **D** total protein ubiquitination and **E** LC3-I and -II and the II/I ratio (*n* = 6/group). **F** Representative blots are shown; immunoblot values were normalized to total protein and expressed relative to young chow controls. Results are reported as mean ± SEM

Given that we observed a similar effect of both aging and diet on metabolic parameters but no age-associated differences in lean mass, we then asked whether the changes accompanying aging and HFD-induced obesity were associated with alterations to muscle mass. Furthermore, we also asked whether HFD-induced obesity and aging would result in similar or distinct changes to muscle fiber size. We measured muscle weight in both non-weight-bearing (TA) and weight-bearing (Plantaris, Gastrocnemius, Soleus) muscles of the hindlimb. Expectedly, we observed a main effect of age for reduced muscle weight in all hindlimb muscles (Fig. 2A). When compared to young diet-matched controls, average muscle mass was lower in the TA (− 17%), soleus (− 16%), plantaris (− 14%), and gastrocnemius (− 19%) in aged chow-fed animals. In contrast to aging, there was a main effect of HFD to enhance muscle mass. Interestingly, HFD-fed aged mice with obesity exhibited a much smaller difference in average muscle mass in the gastrocnemius (− 5%) and plantaris (− 1%) muscles as well as a greater average muscle mass of the soleus (+ 6%) when compared to young mice on a chow diet. In contrast to the changes in weightbearing plantarflexor muscle mass, HFD-fed aged mice exhibited a greater deficit in TA muscle mass (− 8%) compared to young lean controls. Young animals fed the HFD had the greatest average muscle mass when compared to chow-fed young controls in the soleus (+ 25%), plantaris (+ 23%), and gastrocnemius (+ 14%) muscles. Notably, when compared to HFD-fed young mice, HFD-fed aged mice still displayed an average muscle mass that mirrored the deficits in chow-fed animals in the TA (− 11.5%), soleus (− 19%), plantaris (− 24%), and gastrocnemius (− 19%) muscles. These data imply that age-related muscle atrophy is readily observed between groups of similar body composition, but that obesity in aged mice may obscure the aging-associated reduction in muscle mass of weight-bearing muscles when compared to lean young controls. In order to determine how HFD-induced obesity and aging may influence muscle size at the level of the myofiber, we employed immunohistochemical staining of dystrophin in cross sections of the TA muscle (Fig. 2B). We chose to compare myofiber cross-sectional area (CSA) between groups in the TA because it would be influenced by aging and the obesogenic milieu, but not by the anabolic stimulus of a large change in mechanical loading with greater body weight. As expected, group comparisons revealed that the TA muscle exhibited a statistically significant reduction in average fiber CSA in chow-fed aged mice when compared to chow-fed young mice (1541 $\pm$ 80 µM^2^ vs. 1874 $\pm$ 79 µM^2^; *p* = 0.0137) (Fig. 2C). However, HFD-fed aged mice did not exhibit significantly lower fiber CSA (1739 $\pm$ 60 µM^2^) when compared to either chow-fed young (*p* = 0.5234) or aged animals (*p* = 0.2127) or HFD-fed young animals (1789 $\pm$ 52 µM^2^; *p* = 0.9558). Fiber minimum feret diameter tended to follow a similar pattern, with aged animals on a chow diet exhibiting significantly lower fiber feret diameter when compared to young chow (*p* = 0.0031) and HFD-fed animals (*p* = 0.0133) (Fig. 2D). In contrast, aged animals fed a HFD did not differ in minimum feret diameter from any other group. We also measured the cumulative fiber distribution between groups (Fig. 2E) and observed a leftward shift in the TA fiber distribution of chow-fed aged versus chow-fed young animals, which was readily apparent as a reduction in fiber CSA at the 50 th (1821 $\pm$ 185 µM^2^ vs. 1479 $\pm$ 170 µM^2^; *p* = 0.0092) and 75 th percentile (2462 $\pm$ 242 µM^2^ vs. 2017 $\pm$ 263 µM^2^; *p* = 0.0057) (Fig. 2F). HFD-fed aged mice did not exhibit significantly lower TA CSA than chow-fed young mice at the 50 th (1821 $\pm$ 185 µM^2^ vs. 1696 $\pm$ 163 µM^2^; *p* = 0.5718) or 75 th percentile (2462 $\pm$ 242 µM^2^ vs. 2229 $\pm$ 168 µM^2^; *p* = 0.2227), indicating that HFD-fed aged animals exhibited a rightward shift in their fiber distribution. Taken together, these data suggest that aging reduces, while HFD-induced obesity enhances muscle mass in weightbearing plantarflexor muscles. Furthermore, while the effect of aging is readily apparent, aged obese mice exhibit a shift towards larger muscle mass. Interestingly, despite these changes in muscle size, we did not detect an effect of diet on the phosphorylation of AKT, mTORC1, or RPS6 in the TA muscle, but we did detect a main effect of age for enhanced phosphorylation of mTOR (*p* = 0.0389) and RPS6 (*p* = 0.0412) (Fig. 3A–C). We did not detect a change in total protein ubiquitination in the TA muscle (Fig. 3D) but did detect a main effect of age for elevated LC3-I (*p* = 0.032) without main effects for LC3-II or LC3-II/I ratio (Fig. 3E).

### Skeletal muscle lipid content is exacerbated by aging in HFD-fed mice

**Fig. 4:**
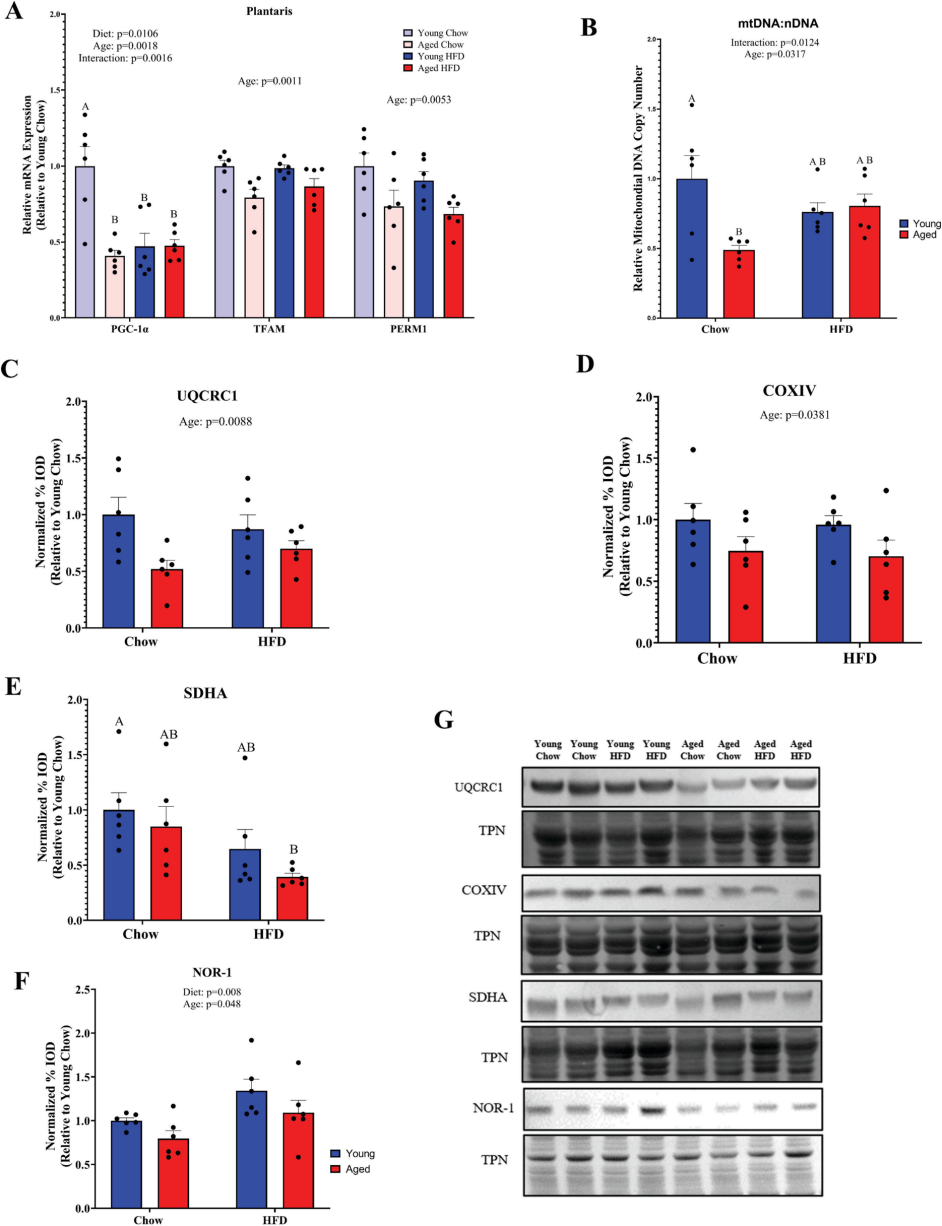
Alterations to mitochondrial content by aging and obesity. **A** Analysis of gene expression using qPCR (HPRT was used as a housekeeping gene) in the plantaris muscle for genes that regulate mitochondrial biogenesis and function. **B** Measurement of the mitochondria DNA (mtDNA) to nuclear DNA (nDNA) ratio in the quadriceps muscle. Mitochondrial protein abundance in the plantaris muscle for components of the electron transport system including **C** UQCRC1, **D** COXIV, and **E** SDHA as well as **F** expression of the nuclear receptor NOR-1. **G** representative blots. Statistical significance was set to *p* < 0.05. Different letters (A, B, C) denote significant differences between groups. Results are reported as mean ± SEM

We next asked whether obesity and aging result in similar changes to mitochondrial content and function. Interestingly, gene expression of the master regulator of mitochondrial biogenesis PGC-1α [34] was significantly reduced in the plantaris muscle by aging in the chow diet group without further repression in HFD-fed aged animals (Fig. 4A). We also observed a main effect of age, but not diet, to reduce expression of TFAM and the striated muscle regulator of mitochondrial function PERM1 [35, 36] (Fig. 4A). TFAM is crucial for mtDNA integrity and maintenance [37]. Given the aging-associated repression of genes involved in mitochondrial biogenesis, we tested whether this resulted in diminished mitochondrial volume as measured by the mtDNA:nDNA ratio in the quadricep muscle (Fig. 4B). We observed both a main effect of age and a significant interaction between our groups. Relative mitochondrial volume, as measured by the mtDNA:nDNA ratio, was 51% lower (*p* = 0.0094) in aged mice on a chow diet compared to chow-fed young mice. However, neither mtDNA:nDNA in HFD-fed aged mice (− 20%, *p* = 0.54) nor HFD-fed young mice (− 24%, *p* = 0.36) differed significantly from chow-fed young mice. It is worth noting that while not statistically significant, HFD-induced obesity increased relative mtDNA content in aged animals (+ 56%, *p* = 0.15) but led to a decrease in young animals compared to chow-fed age-matched controls. In line with the findings that measures of mitochondrial volume are altered by age, we observed a significant effect of age in the plantaris for reduced mitochondrial electron transport system components UQCRC1 (*p* = 0.0088) and COXIV (*p* = 0.0381) (Fig. 4C, D). Interestingly, pairwise comparisons revealed a significant reduction in the complex II component SDHA in HFD-fed aged mice when compared to chow-fed young mice (− 61% *p* = 0.0132) (Fig. 4E). We also observed in the plantaris a main effect for age to decrease (*p* = 0.048), and HFD to increase (*p* = 0.008) protein abundance of the exercise responsive nuclear receptor NOR-1 (Fig. 4F). Importantly, NOR-1 is associated with oxidative metabolism in skeletal muscle [38]; however, its relevance to aging and obesity is poorly understood.

**Fig. 5:**
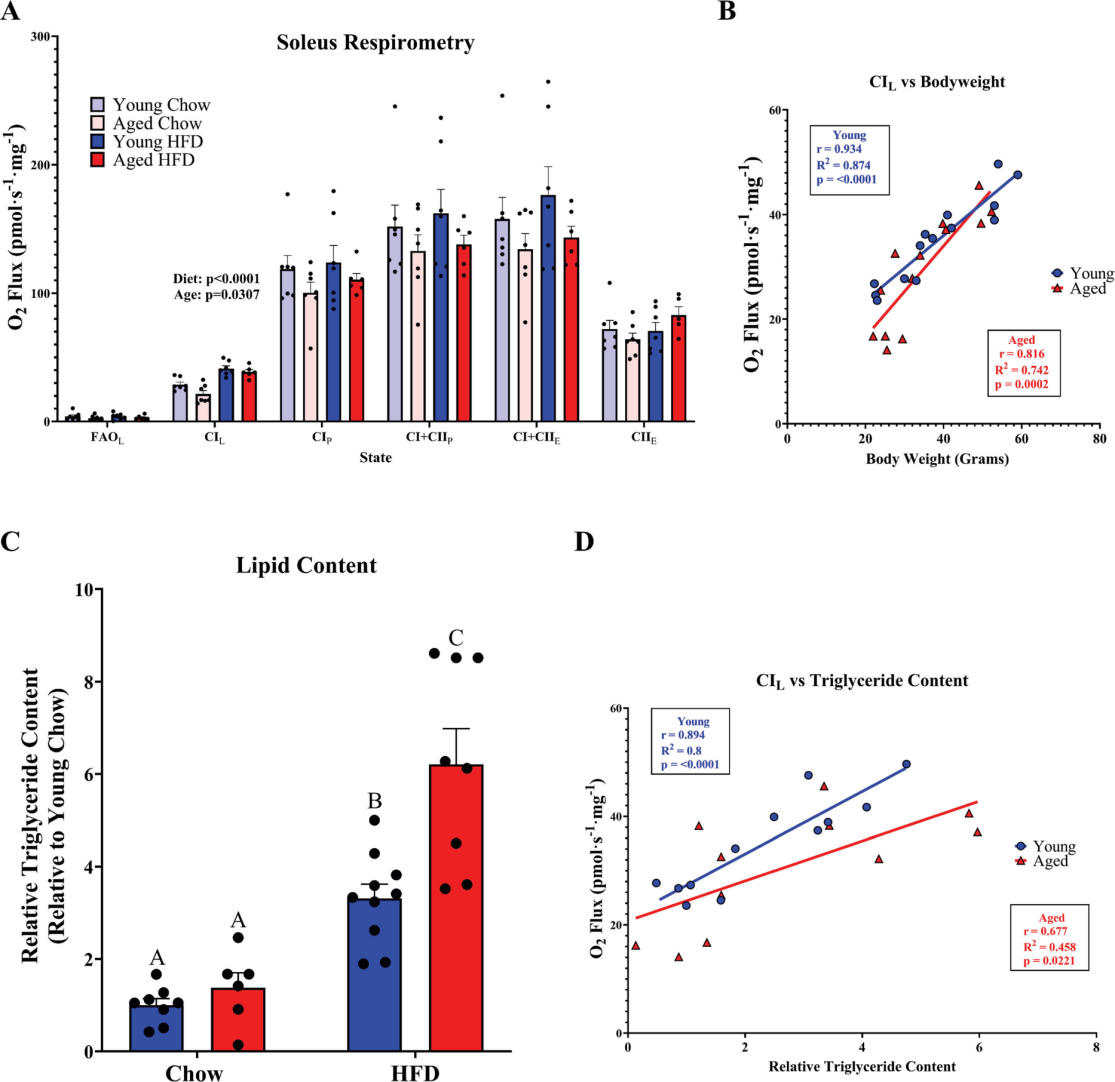
Aging and obesity distinctly regulate mitochondrial proton leak but have additive effects on muscle triglyceride content. **A** High-resolution respirometry of mouse soleus permeabilized fibers. States measured were (1) fatty acid oxidation mediated leak using palmitoylcarnitine and malate (FAO_L_), (2) leak respiration after addition of complex I substrates glutamate, pyruvate, and malate (CI_L_), (3) OXPHOS respiration after addition of ADP (CI_P_), (4) OXPHOS respiration after addition of succinate (CI + CII_P_), (5) respiration after uncoupling of the electron transfer system during stepwise addition of CCCP (CI + CII_E_), and (6) uncoupled complex II driven respiration after inhibition of complex I and fatty acid oxidation with rotenone (CII_E_). **B** Correlation of CI_L_ and body weight. **C** Skeletal muscle triglyceride content in the quadricep muscle normalized to protein content and plotted relative to young chow values. **D** Soleus CI_L_ was correlated to quadricep triglyceride content. Statistical significance was set to *p* < 0.05. Different letters (A, B, C) denote significant differences between groups. Results are reported as mean ± SEM

Given the pronounced effects of aging on the expression of regulators of mitochondrial biogenesis, mtDNA content, and mitochondrial protein abundance, we then asked whether the effects of aging and HFD-induced obesity on mitochondrial function are different. Mitochondria are the primary site of ATP production and lipid oxidation. Therefore, deficits in mitochondrial volume or function have profound implications for substrate oxidation. Using high-resolution respirometry, we observed a stark effect of diet (*p* < 0.0001) to elevate complex I driven mitochondrial proton leak (CI_L_) (Fig. 5A). We also observed a main effect of age (*p* = 0.0307) for reduced CI_L_. Uncoupling proton flow from ATP synthesis can be a mechanism to lessen reductive stress during nutritional overload by allowing an alternate way for proton backpressure in mitochondria to be alleviated independent of ATP synthase. Indeed, uncoupling can mitigate ROS production, but it can also, through competition with ATP synthase, allow for greater energy expenditure as is the case for brown adipose tissue. Skeletal muscle mitochondrial proton leak constitutes a significant portion of resting energy expenditure and is positively associated with weight loss in humans [39]. Interestingly, we found that in both young (*r* = 0.894, *p* < 0.0001) and aged (*r* = 0.677, *p* = 0.0002) animals, skeletal muscle mitochondrial proton leak was highly positively associated with body weight (Fig. 5B). Given that in aged animals we detected diminished mitochondrial volume, protein abundance, and impairments in whole body metabolism, we then asked whether the changes in mitochondrial volume and function with aging were associated with changes in skeletal muscle lipid content. Measuring quadricep triglyceride content revealed that at baseline, lean chow-fed young and aged mice did not differ from each other in skeletal muscle triglyceride content (*p* = 0.8441) (Fig. 5C). As expected, HFD-fed young mice displayed significantly more muscle triglyceride content than either chow-fed young (*p* = 0.001) or aged (*p* = 0.0046) mice. However, HFD-fed aged mice exhibited an exacerbated response to obesity and had greater muscle triglyceride content than chow-fed young (*p* = 0.0016), aged (*p* = 0.0015), and HFD-fed young animals (*p* = 0.0359). Interestingly, despite HFD-fed aged animals having a similar bodyweight and body composition to HFD-fed young animals, muscle from aged obese animals contained significantly greater triglyceride content than young diet-matched counterparts. We then evaluated the Pearson correlation coefficient for CI_L_ and relative muscle triglyceride content and found a positive association in both young (*r* = 0.894; *p* < 0.0001) and aged animals (*r* = 0.677; *p* = 0.0221) between CI_L_ and muscle triglyceride content (Fig. 5D).

### Effects of aging and obesity on the muscle transcriptome

HFD-induced obesity resulted in stark changes in muscle mass and mitochondrial respiration, which can be divergent from the effects of aging. Thus, we performed 3′ RNA-Seq on gastrocnemius muscle RNA to determine how HFD-induced obesity altered the transcriptome of young and aged animals. Visually, we observed prominent separation between clusters of young chow-fed and aged HFD-fed animals on our PCA plot (Supplementary Fig. 1A). Our bioinformatic analysis revealed the top differentially expressed genes (DEGs) altered between each condition (Supplementary Fig. 1B-F). Notably, there were several unique DEGs when comparing the top genes altered between aged chow-fed and aged HFD-fed animals relative to young chow-fed animals. These results were mirrored in volcano plots (Supplementary Fig. 2A-E), which exhibited a greater number of DEGs between HFD-fed aged than chow-fed aged mice when compared to young chow-fed mice (top 5 most upregulated and downregulated DEGs annotated). Notably, among the genes with the highest FC, we observed Jchain, KRT18, and Sln to be upregulated in chow-fed and HFD-fed aged mice when compared to chow-fed young mice. Sln (sarcolipin) is known to regulate SERCA pump activity and ATP hydrolysis [40] and appears to be transcriptionally upregulated in aged muscle regardless of body composition. Remarkably, relative to chow-fed young animals, we observed that four of the five most downregulated DEGs were conserved in HFD-fed mice groups regardless of age. These genes included CEBPD, DDIT4, Depp1, and Doc2b. Gene ontology (Table 1) and pathway analysis (Supplementary Fig. 3) revealed an enrichment for DEGs associated with metabolic and inflammatory pathways between groups. While chow-fed aged animals exhibited DEGs enriched for the biological process terms “Immune system processes,” “Neutrophil migration,” and “Leukocyte migration” relative to chow-fed young animals, aged HFD-fed animals were enriched for DEGs associated with the biological processes “Response to external stimulus,” Immune system process,” and “Response to biotic stimulus.” These data indicate that aged mice, regardless of body composition, exhibit a skeletal muscle transcriptome characterized by immune-associated gene expression when compared to young lean animals; however, the biological processes associated with these genes may be altered by body composition. Interestingly, when comparing HFD-fed young to chow-fed young animals, we observed an enrichment for genes belonging to the biological processes “Fat cell differentiation” and “Vasculature development.” However, when comparing enriched biological processes between chow-fed aged and HFD-fed aged animals, we observed an enrichment for the terms “Biological Process Involved in Interspecies Interaction Between Organisms” and “Response to Biotic Stimulus.” These data suggest that HFD-induced obesity modulates genes associated with the immune system in aged mice to a greater degree than in young mice.

**Table 1:** Bioinformatic analysis of RNA-Seq data reveals the overrepresented biological processes, cellular compartments, and molecular functions between each group comparison

Gene ontology	Biological processes	Cellular component	Molecular function
Aged chow vs young chow	• Leukocyte migration • Immune system processes • Neutrophil migration • Response to stimulus • Regulation of immune system processes	• Extracellular space • Platelet alpha granule • Cell periphery • Extracellular region • Actin cytoskeleton	• Neurotransmitter binding • Signaling receptor regulator activity • Protein binding • Acetylcholine binding • Receptor ligand activity
Young HFD vs young chow	• Response to stimulus • Fat cell differentiation • Vasculature development • Multicellular organism development • Cellular response to stimulus	• Collagen-containing extracellular matrix • External encapsulating structure • Extracellular matrix • Extracellular region • Extracellular space	• Extracellular matrix structural constituent • Collagen binding • Protein binding • Extracellular matrix structural constituent conferring tensile strength • Sulfur compound binding
Aged HFD vs young chow	• Response to external stimulus • Immune system process • Response to biotic stimulus • Response to external biotic stimulus • Response to other organism	• Cell periphery • Plasma membrane • Extracellular region • Extracellular space • External side of plasma membrane	• Protein binding • Glycosaminoglycan binding • Sulfur compound binding • Cell adhesion molecule binding • Binding
Aged HFD vs young HFD	• Immune system process • Immune response • Response to external biotic stimulus • Response to other organism • Response to biotic stimulus	• External side of plasma membrane • Side of membrane • Cell surface • Plasma membrane • Cell periphery	• Transmembrane signaling receptor activity • Signaling receptor activity • Molecular transducer activity • Immune receptor activity • Antigen binding
Aged HFD vs aged chow	• Biological process involved in interspecies interaction between organisms • Response to biotic stimulus • Response to external biotic stimulus • Response to other organism • Response to external stimulus	• Extracellular region • Extracellular space • Host cellular component • Symbiont-containing vacuole • Symbiont-containing vacuole membrane	• Peptide antigen binding

**Fig. 6:**
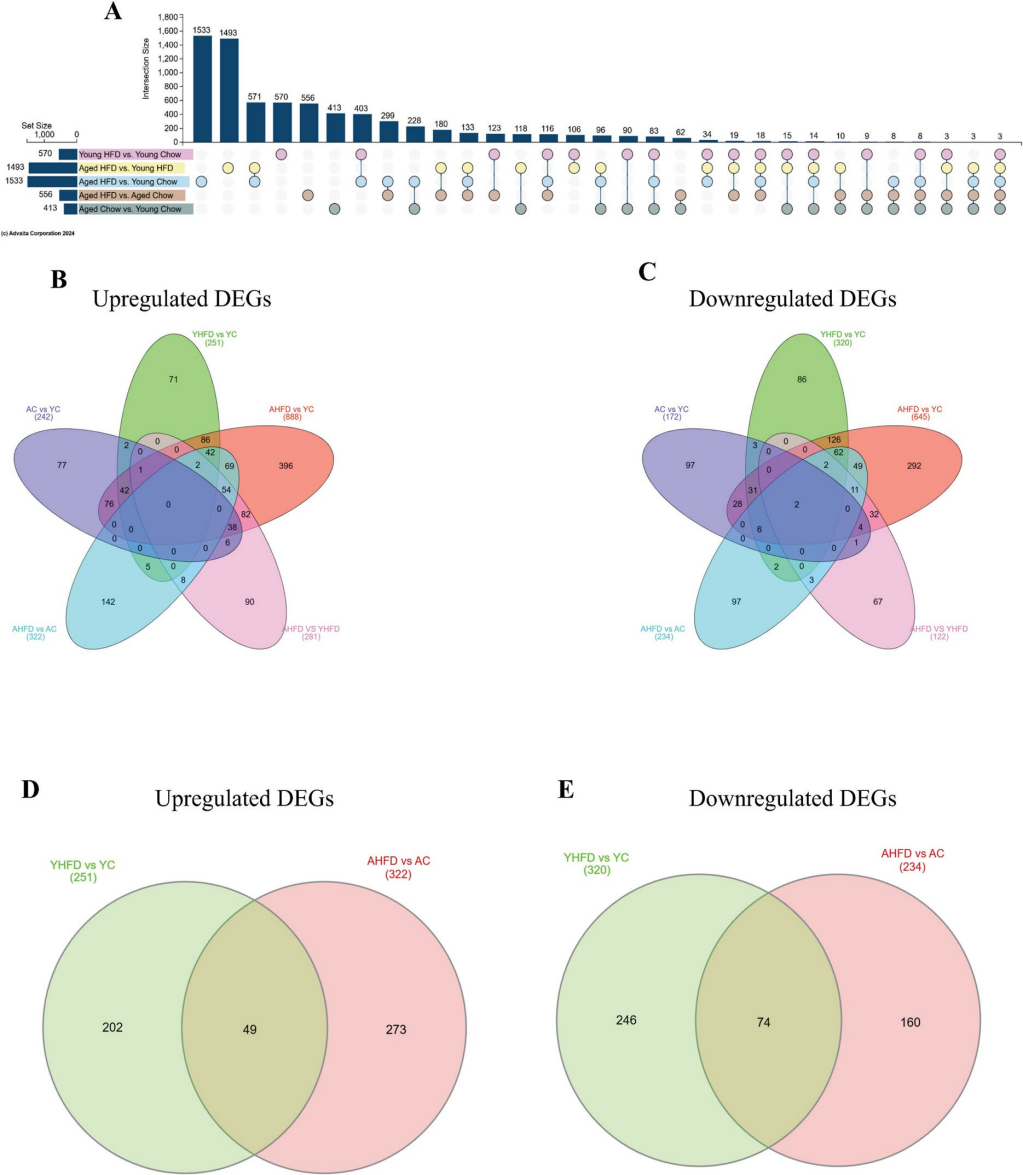
Aged animals exhibit unique skeletal muscle gene changes when fed HFD. **A** Upset plot illustrating intersections between group comparisons. Venn diagrams indicating common and distinct **B** upregulated and **C** downregulated DEGs between groups. Venn diagrams comparing the impact of HFD induced obesity on **D** upregulated and **E** downregulated DEGs between young and aged animals. YC, young chow; AC, aged chow; YHFD, young HFD; AHFD, aged HFD

To further investigate how transcriptomic changes differ between young and aged animals on HFD, we analyzed the unique gene changes between each condition. Utilizing an Upset plot to visualize gene changes revealed that the largest intersection between comparisons was detected when comparing HFD-fed aged animals to either group of young adult animals (Fig. 6A). Importantly, of the 1533 genes that were detected as being differentially expressed between HFD-fed aged and chow-fed young animals, only 228 (15%) were also differentially expressed between chow-fed young and aged animals. These data suggest that HFD-induced obesity produces robust and unique transcriptional changes in muscles of aged animals. Interestingly, three genes (Dnajb5, Spns2, and Txnip) were differentially expressed in all comparisons. A closer look at the directionality of changes to gene expression reveals that relative to chow-fed young animals, 157/242 (65%) of significantly upregulated genes in chow-fed aged animals were also upregulated in HFD-fed aged animals. However, HFD-fed aged animals exhibited an additional 731/888 (82%) upregulated DEGs (Fig. 6B). Downregulated genes displayed a similar trend (Fig. 6C), with 71/172 (41%) of DEGs downregulated in muscles of chow-fed aged animals relative to chow-fed young animals also being downregulated in muscles of HFD-fed aged animals relative to chow-fed young animals. However, 574/645 (90%) of downregulated genes in the HFD-fed aged animals were not significantly downregulated in chow-fed aged animals. We then contrasted the up- and downregulated genes altered by HFD feeding between young and aged animals to compare the transcriptional response of young and aged animals to HFD-induced obesity. Interestingly, we detected that only 49 DEGs (20% and 18% in young and aged animals, respectively) were similarly upregulated by HFD feeding across ages (Fig. 6D). Furthermore, we observed 74 common downregulated DEGs (30% and 46% in muscles of young and aged animals, respectively) between ages with HFD feeding (Fig. 6E). Interestingly, the difference in DEGs detected between chow-fed young and aged animals (413 DEGs) was much smaller than that of HFD-fed young and aged animals (1493 DEGs). These data imply that HFD-induced obesity results in a robust transcriptional response in aged animals and that the majority of DEGs altered by HFD feeding in aged animals are not differentially expressed with aging or diet independently.

## Discussion

Aging appears to be, thus far, an inevitable biological process characterized by a loss of tissue integrity [41, 42], function [43], and resilience to insult [42, 44]. While great strides have been made to extend the human lifespan, the presence of chronic disease and aging-associated illness diminishes quality of life in the elderly. Skeletal muscle health is a prime determinant of quality of life due to its crucial role in locomotion and whole-body metabolism. While factors intrinsic to the aging process such as telomere shortening [45, 46], DNA damage and apoptosis [47], senescence [48, 49], and loss of proteostasis [5] are known to induce tissue dysfunction, we must expand our understanding of how extrinsic factors (diet, physical activity, environmental factors) alter the trajectory of aging. Broadening our knowledge of how these extrinsic factors may influence several aspects of aging is crucial before effective and innovative interventions can be employed. The intersection of an increasingly aging population with an obesity epidemic has gained clinical attention [15, 50, 51], as more elderly people become obese. Importantly, several preclinical studies that investigate the effects of aging frequently utilize aged mice that are considerably heavier than young controls [19, 20, 23]. How obesity may alter and influence aspects of aged muscle (muscle atrophy, mitochondrial content and function, altered gene expression) is a poorly addressed gap in the literature. Our study investigated the impact of HFD-induced obesity and aging on skeletal muscle metabolism and gene expression. To our knowledge, this is the first study to directly compare the skeletal muscle transcriptomic response to aging and obesity separately and together.

Our study reveals several important findings regarding the interaction between aging and obesity. Firstly, we found that young and aged mice become obese when fed a HFD at a similar rate and do not differ significantly from each other in either body weight or body composition. The similar rate of weight gain between young and aged HFD-fed animals is interesting because we detected an effect of age to reduce food intake, which was reflected in a loss of body weight in chow-fed aged mice. As expected, we found an effect of age to decrease muscle wet weight in all muscles measured, which is consistent with an aging-associated loss of skeletal muscle. However, the observed effect of obesity on enhancing muscle wet weight was not expected. The effect of obesity on muscle mass was primarily observed in the gastrocnemius, plantaris, and soleus muscles but not the tibialis anterior, suggesting that the muscle growth may have been driven by increased mechanical loading of the weight-bearing plantarflexor muscles. These findings have important implications for the study of age-associated muscle loss. Although the effect of age on muscle mass is still readily observed between animals of similar body weights, comparisons between young lean animals and aged obese animals obfuscate the aging-associated loss of muscle. Our investigation of the Akt-mTORC1 signaling pathway and our measurements of ubiquitination and autophagy did not explain the obesity-associated muscle growth in aged or young animals. It is possible that the pathways involved in the regulation of muscle mass by obesity may not be readily detectable in our animals because they were fasted overnight and thus may not represent changes that occur after feeding. Alternatively, it is possible that the pathways regulating muscle mass in response to HFD feeding may be active early on during the rapid gain of body weight at the start of the diet but not at the timepoint that we harvested tissues. Alternatively, it is also possible that other pathways are driving the increase in muscle weight we observed. While our measurement of mTORC1 and its downstream target RPS6 did not explain the obesity-associated muscle growth, these findings suggest a moderate activation of mTORC1 signaling in our aged animals. Interestingly, we found that aging was associated with elevated LC3-I but not LC3-II or an altered LC3-I/II ratio. Given that muscle atrophy is driven, in large part, by a reduction in myofiber size, we also examined fiber CSA in the TA muscle. We chose the TA muscle because it is not a weight-bearing muscle. This allowed us to examine the effects of aging and the obesogenic milieu without the background of the anabolic/growth stimulus from elevated muscle loading resulting from the greater body weight on a weight-bearing muscle. Interestingly, we found significant atrophy of muscle fibers in aged chow-fed mice but not in aged or young HFD-fed mice. Investigating the cumulative frequency of fiber sizes revealed that HFD-fed aged mice exhibited a rightward shift of the cumulative frequency curve as they did not differ in median fiber size from young chow-fed animals. Given the advanced starting age of our mice (23–27 months) and the short duration of our diet (14 weeks), it is likely that the coincidence of obesity is reversing, instead of preventing, the loss of muscle mass with age.

Muscle mass is a crucial determinant of whole-body metabolic health; thus, we sought to define if aging and obesity have divergent effects on metabolism. At the level of the whole body, relative VO_2_, VCO_2_, and RER were impacted by aging and obesity in a similar way, suggesting a potentially additive effect. Due to the integral role of mitochondria in metabolic processes, we turned our attention to the volume and function of skeletal muscle mitochondria. We observed repressed expression of several important regulators of mitochondrial biogenesis and function with aging (PGC-1α, TFAM, NOR-1, and PERM1), but we only observed an effect of diet to reduce PGC-1α expression. In line with these findings, mitochondrial DNA content was potently repressed by aging, but surprisingly, we did not observe an effect of diet.

Mitochondrial proton leak can be a mechanism to alleviate reductive stress during nutritional overload by allowing an alternate way for proton backpressure in mitochondria to be alleviated independent of ATP synthase and ATP demand. Leak is known to be activated by fatty acids [52] and is mediated by changes in mitochondrial lipid composition [53], uncoupling proteins [54, 55], and ANT1 [56]. We found that HFD-induced obesity greatly elevated complex I-driven mitochondrial proton leak. Our finding that aged mice have exacerbated skeletal muscle lipid accumulation in response to HFD, agrees with work from others, indicating that aged mice exhibit extensive lipidome remodeling on HFD [57]. However, whether the elevated muscle triglyceride content is due to elevated intramuscular or intermuscular lipids is poorly understood. Furthermore, whether the accumulation of lipids in aged obese muscle is due to excessive import or impaired oxidation remains to be conclusively determined. Our data would suggest that aged animals may exhibit a minor defect in the induction of complex I-driven leak by HFD. It may be possible that aged animals do not elevate leak as readily as young animals, leading to a steady accumulation of lipids over time. Our finding that leak is tightly associated with bodyweight in both young and aged animals would suggest that elevating leak may be a protective mechanism in the context of substrate overload, as humans who exhibit greater muscle mitochondrial proton leak have more success with losing weight [39]. It may also be possible that elevated lipid content may be altering the transcriptome of aged animals.

Our bioinformatic analysis of how aging and obesity alter the muscle transcriptome suggests that HFD-induced obesity leads to extensive alterations in the whole muscle gene expression of aged animals. These findings are important to inform future transcriptomic studies that may compare aged obese animals to young controls, as obesity drastically increased the number of DEGs that would be observed when compared to young chow-fed controls and produced little overlap in the DEGs found when comparing between chow-fed young and aged animals. HFD-feeding led to alterations in genes associated with the “PI3 K-AKT signaling pathway” and “FOXO signaling pathway” in young animals. However, in aged animals, HFD-feeding resulted in an enrichment of DEGs belonging to the “antigen processing pathway,” “AMPK signaling pathway,” and “PPAR signaling pathway.” The information theory of aging indicates that apart from chronological age, molecular changes such as the loss of epigenetic information can be a primary contributor to biological aging [58]. Central to this paradigm is the hypothesis that environmental factors, such as diet and physical activity, can influence the accumulation of cellular damage that modulates the epigenome. Indeed, epigenetic biomarkers of aging, such as DNA methylation, are well correlated to lifespan, associated with several aging-related conditions [59], and influenced by lifestyle factors such as diet and exercise [60]. Additionally, the aging-related upregulation of the interferon pathway is associated with chromatin remodeling at endogenous retroviral sequences [61]. While both groups of aged mice exhibited an enrichment for genes associated with immune function compared to chow-fed young mice, HFD-feeding resulted in the enrichment of genes associated with the “Viral protein interaction” and “Viral myocarditis.” It is also interesting to note that when comparing HFD-fed aged animals to either chow-fed or HFD-fed aged animals, our bioinformatic analysis revealed that several inflammation-associated targets inhibited by GPSM1, GPSM2, and GPSM3 were upregulated (Supplementary Fig. 4). Notably, GPSM1 is highly associated with type 2 diabetes and is known to control inflammatory signaling [62].

There are several limitations in our study. Utilizing HFD-feeding as our model of obesity does not fully recapitulate how obesity may develop in aged persons or the progression of obesity that occurs from middle-aged to elderly. Furthermore, given that we harvested tissues after an overnight fast, it is possible that mitochondrial bioenergetics, as well as anabolic and catabolic signaling, may differ in the fed state and be more representative of muscle mass changes we observed. Indeed, it is also possible that throughout the study, young and aged animals may have differed substantially in their rate of adaptation to obesity. Our study was not designed to investigate if changes that occur with obesity may happen at different rates between young and aged animals. It is unknown if young animals increase mitochondrial proton leak in skeletal muscle more readily or sooner than aged animals in the context of obesity. Additionally, while we only measured changes in fiber size in the TA muscle and therefore we could not entirely rule out that changes in muscle wet weight in the plantarflexor muscles were due to changes in non-muscle tissue such as intermuscular fat, collagen, or inflammation.

There remain a number of new unanswered questions that serve as future directions and can enhance the current findings. Firstly, whether enhancing mitochondrial function and volume can mitigate lipid accumulation in the aged muscle remains to be adequately addressed. Chemical uncouplers have shown promise in treating sarcopenic obesity, presumably through the activation of mitochondrial quality control pathways [63]. Whether chemical uncouplers can mitigate the alterations to lipid content and lipid species accumulation in aged obese skeletal muscle, and therefore mitigate inflammatory signaling, remains to be tested. Although we did not test systemic markers of inflammation, it is known that skeletal muscle is the largest cytokine producing organ in the body. Aging is associated with chronic inflammation [9, 64], which is an important contributor to tissue dysfunction. Therefore, it will be crucial in future investigations to explore the underlying mechanisms and strategies to mitigate how aging and obesity together may influence inflammation in skeletal muscle and whether this synergism is due to epigenetic changes. Furthermore, whether changes to fiber size were altered by obesity in a fiber type-dependent manner between young and aged animals is a gap in our understanding. While it is known that aging is associated with atrophy of type II fibers, how this relationship is influenced by obesity is poorly understood. Taken together, our data reveal that aging and obesity have different effects on mitochondrial proton leak and synergize to induce elevated muscle lipid content. Furthermore, our study also addresses the rigor of prior literature and suggests that the body composition of aged animals is an important factor to consider in aging studies.

## Supplementary Information

Below is the link to the electronic supplementary material.

Supplementary file1 Supplementary Figure 1. Top gene changes associated with aging and HFD-induced obesity. A) PCA plot of all four groups. B-F) Heatmaps of the top 30 DEGs between conditions. (JPG 2589 KB)

Supplementary file2 Supplementary Figure 2. Volcano plots of DEGs associated with aging and HFD-induced obesity.  A-E) Volcano plots showing measured genes which passed the FDR threshold of 0.05 in red. The top 5 downregulated and upregulated genes with the largest fold change are annotated between comparisons. (JPG 2507 KB)

Supplementary file3 Supplementary Figure 3. Top pathways altered between conditions. A-E) Chord diagrams showing the top pathways altered between groups and the genes within each pathway that exhibited the largest fold change. (JPG 3343 KB)

Supplementary file4 Supplementary Figure 4. Gene network displaying a number of DEGs upregulated in HFD-fed aged animals relative to chow-fed young animals that are regulated by GPSM1-3. (JPG 2092 KB)

Supplementary file5 Supplementary Table 1. Table of antibodies used. (DOCX 15 KB)

Supplementary file6 Supplementary Table 2. Table of primers used. (DOCX 14 KB)

## Data Availability

All data required to evaluate the conclusions in this manuscript are available within the manuscript and supporting information. Raw data can be made available from the corresponding author upon reasonable request.
