# Supplementary figures and images for: Impact of sarcopenia and obesity on skeletal muscle size, gene expression, and mitochondrial function
Source: GeroScience. 2025 Jun 12;48(1):1219–40. doi: 10.1007/s11357-025-01726-2 (PMC12972386; doi:10.1007/s11357-025-01726-2)

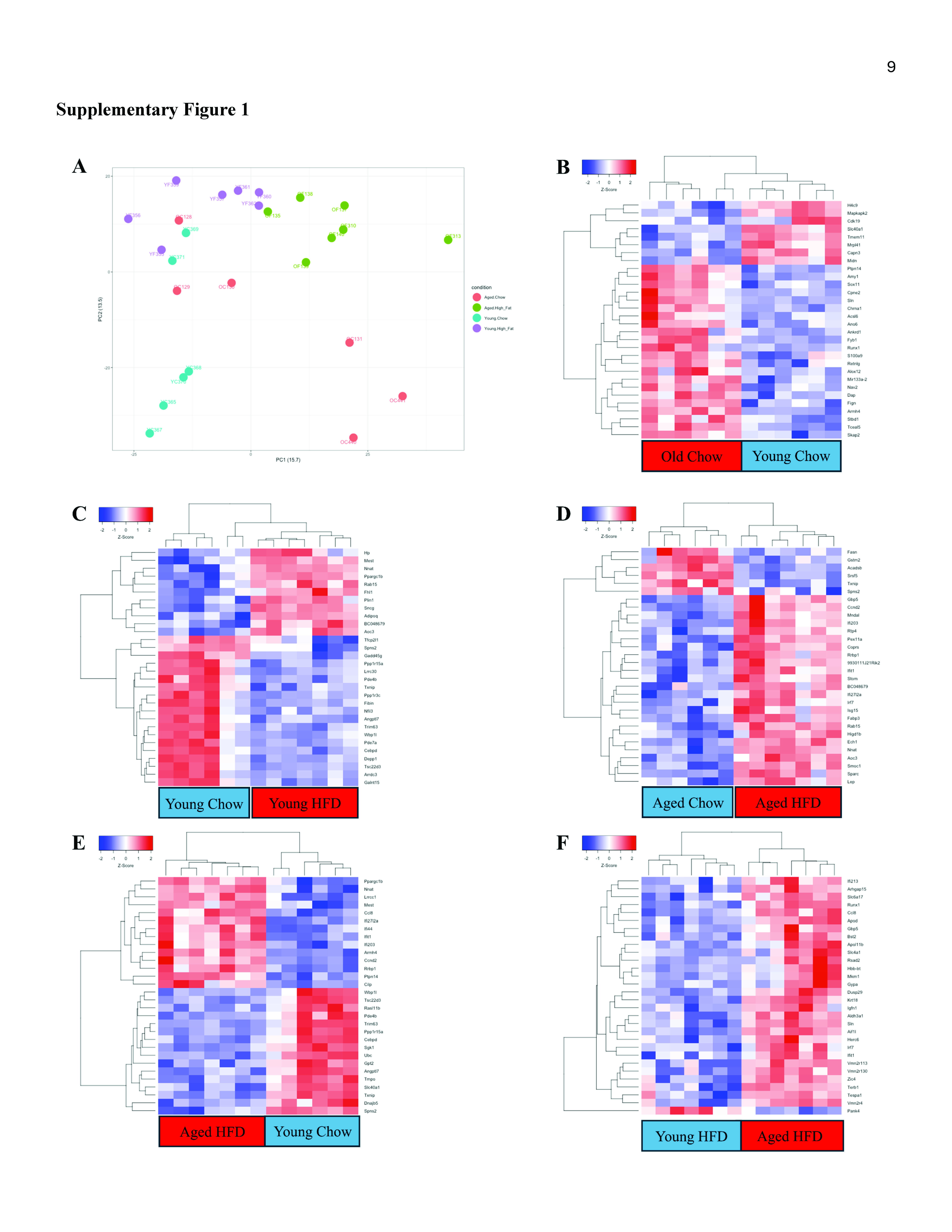

Supplement: Supplementary file 1 — Supplementary file1 Supplementary Figure 1. Top gene changes associated with aging and HFD-induced obesity. A) PCA plot of all four groups. B-F) Heatmaps of the top 30 DEGs between conditions. (JPG 2589 KB) [file 11357_2025_1726_MOESM1_ESM.jpg]

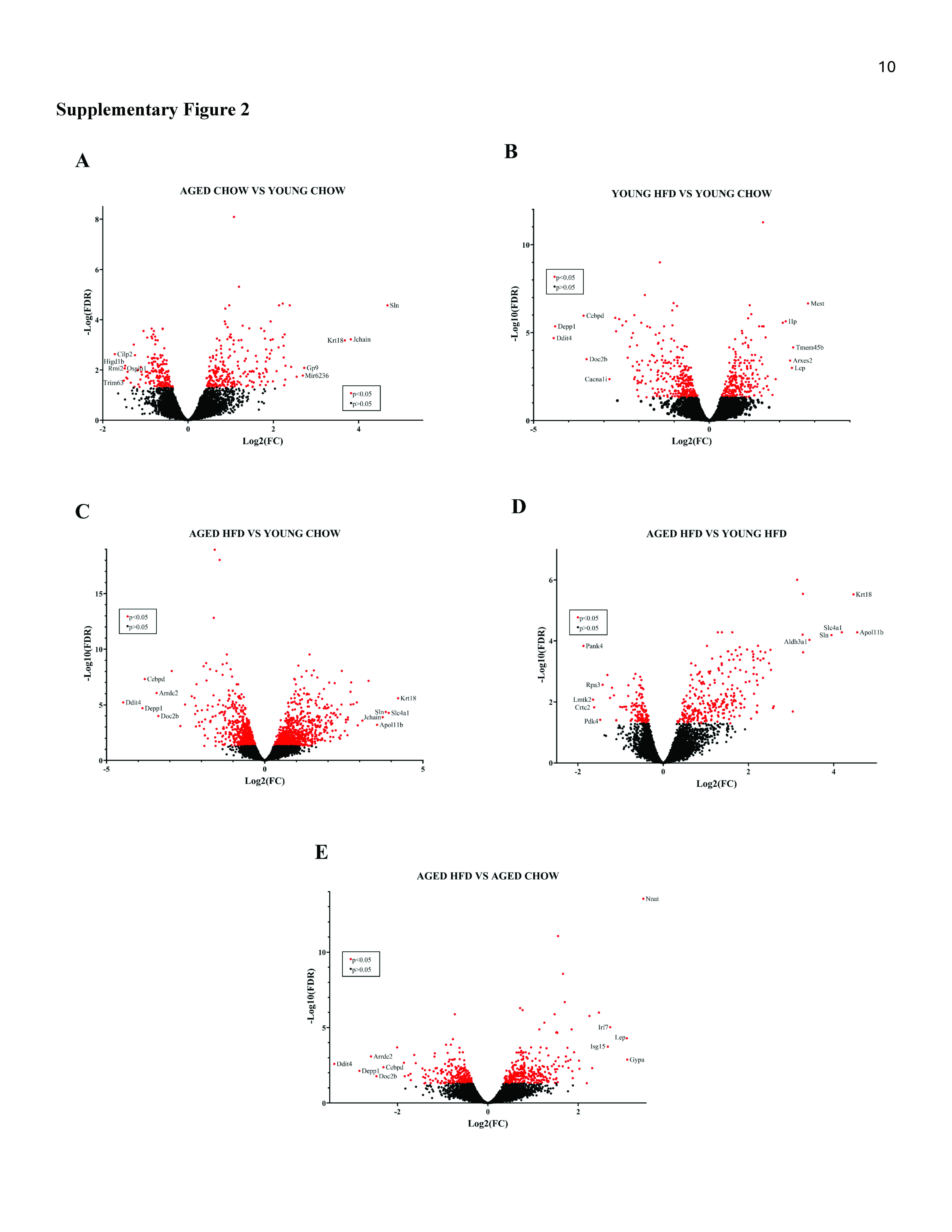

Supplement: Supplementary file 2 — Supplementary file2 Supplementary Figure 2. Volcano plots of DEGs associated with aging and HFD-induced obesity. A-E) Volcano plots showing measured genes which passed the FDR threshold of 0.05 in red. The top 5 downregulated and upregulated genes with the largest fold change are annotated between comparisons. (JPG 2507 KB) [file 11357_2025_1726_MOESM2_ESM.jpg]

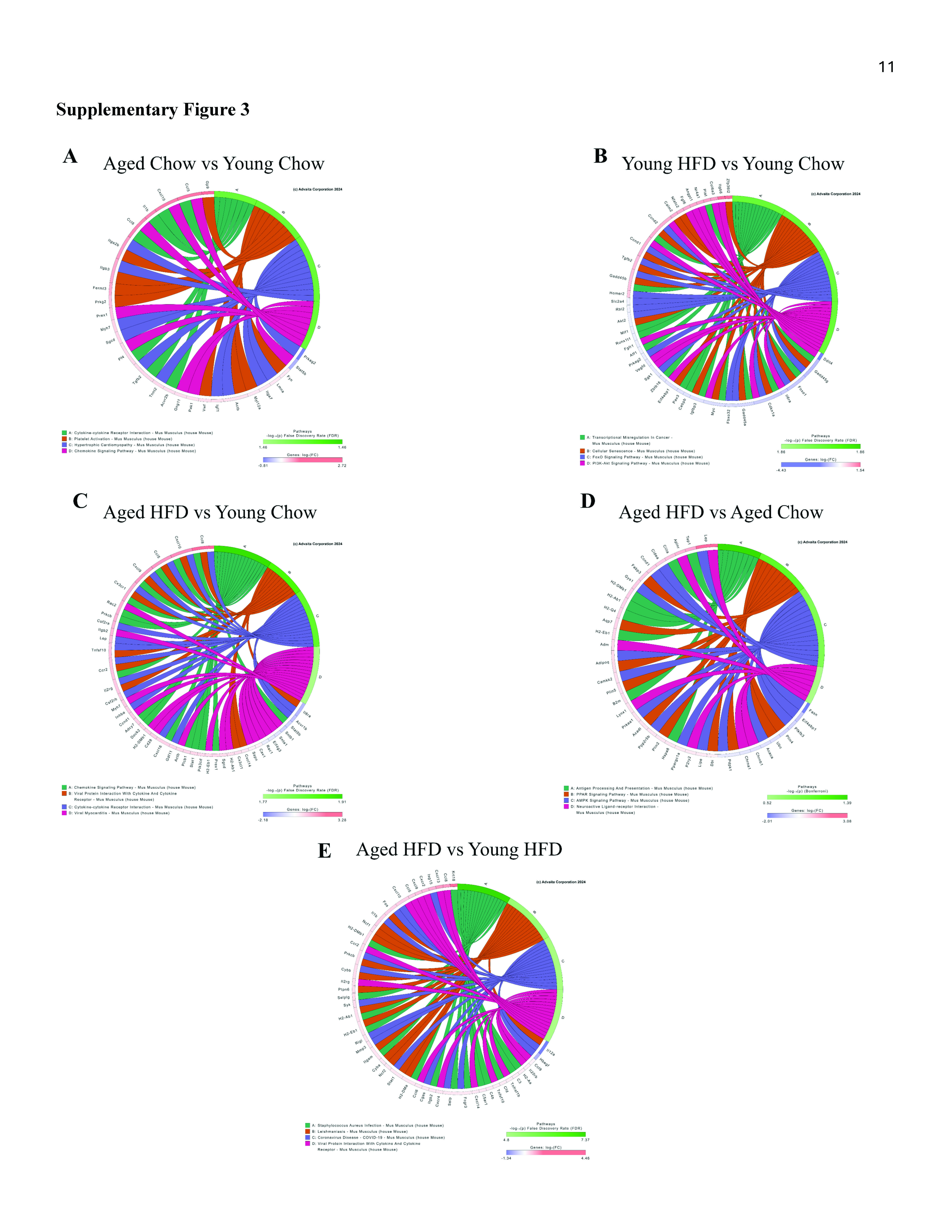

Supplement: Supplementary file 3 — Supplementary file3 Supplementary Figure 3. Top pathways altered between conditions. A-E) Chord diagrams showing the top pathways altered between groups and the genes within each pathway that exhibited the largest fold change. (JPG 3343 KB) [file 11357_2025_1726_MOESM3_ESM.jpg]

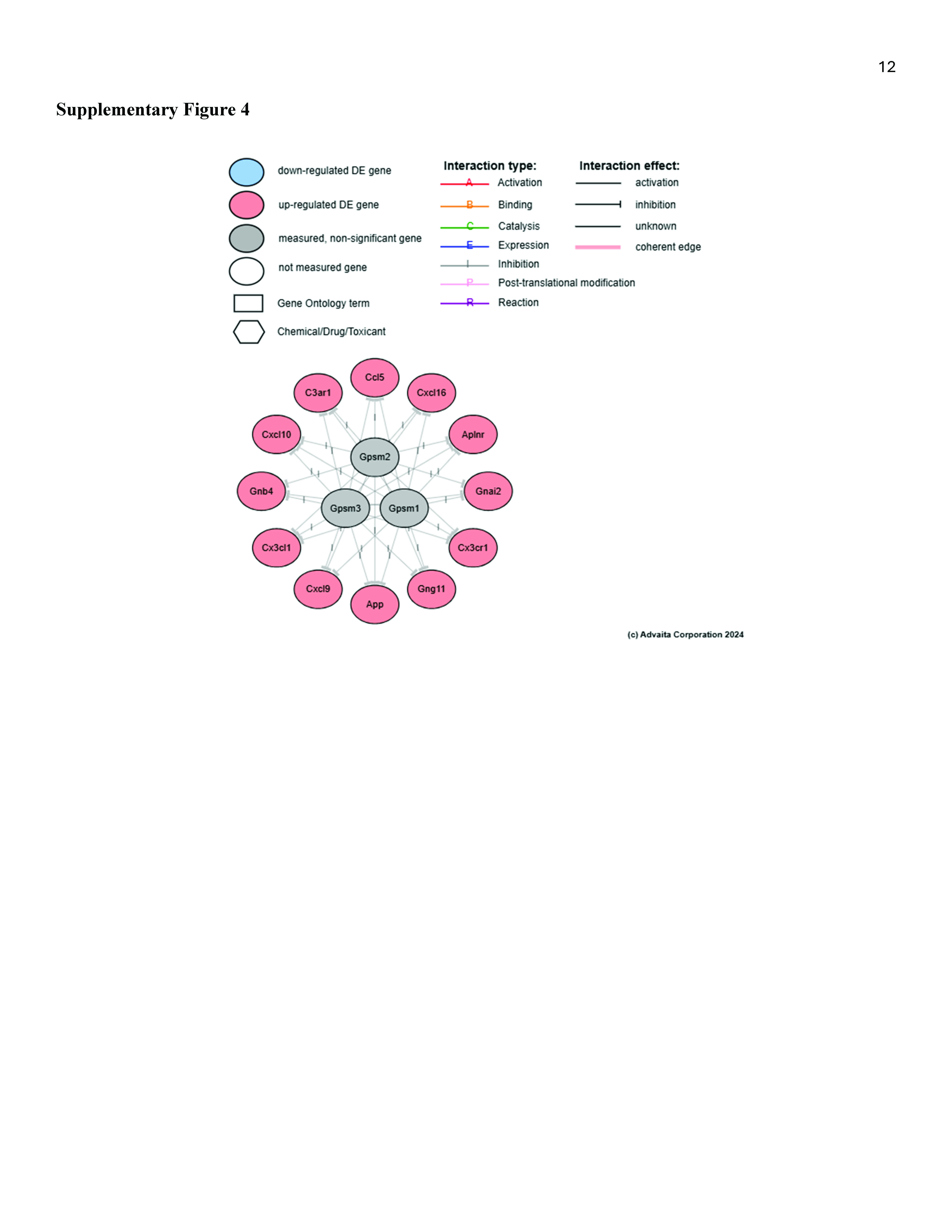

Supplement: Supplementary file 4 — Supplementary file4 Supplementary Figure 4. Gene network displaying a number of DEGs upregulated in HFD-fed aged animals relative to chow-fed young animals that are regulated by GPSM1-3. (JPG 2092 KB) [file 11357_2025_1726_MOESM4_ESM.jpg]
