# Supplementary material for: Impact of sarcopenia and obesity on skeletal muscle size, gene expression, and mitochondrial function
Source: GeroScience. 2025 Jun 12;48(1):1219–40. doi: 10.1007/s11357-025-01726-2 (PMC12972386; doi:10.1007/s11357-025-01726-2)
Supplement: Supplementary file 5 — Supplementary file5 Supplementary Table 1. Table of antibodies used. (DOCX 15 KB) [file 11357_2025_1726_MOESM5_ESM.docx]

Supplementary Table 1

| **Antibody** | **Manufacturer** | **Item#** |
| --- | --- | --- |
| mTOR | Cell Signaling | 2972S |
| Phospho-mTOR (Ser 2448) | Cell Signaling | 2971S |
| S6 | Cell Signaling | 2217S |
| Phospho-S6 (Ser235/236) | Cell Signaling | 62016S |
| Akt | Cell Signaling | 4691T |
| Phospho-Akt (Thr308) | Cell Signaling | 13038S |
| Ubiquitin | Cell Signaling | 58395S |
| LC3 | Cell Signaling | 4108S |
| UCQRC1 | Proteintech | 21705-1-AP |
| COXIV | Cell Signaling | 4850S |
| SDHA | Cell Signaling | 11998S |
| NR4A3 | Sigma-Aldrich | ABE1456 |
| Dystrophin | DSHB | AB_2618170 |
