# Supplementary material for: Impact of sarcopenia and obesity on skeletal muscle size, gene expression, and mitochondrial function
Source: GeroScience. 2025 Jun 12;48(1):1219–40. doi: 10.1007/s11357-025-01726-2 (PMC12972386; doi:10.1007/s11357-025-01726-2)
Supplement: Supplementary file 6 — Supplementary file6 Supplementary Table 2. Table of primers used. (DOCX 14 KB) [file 11357_2025_1726_MOESM6_ESM.docx]

Supplementary Table 2

| **Gene** | **Primer** | **Sequence 5'•3'** |
| --- | --- | --- |
| mtDNA(ND2)  **nDNA (Pecam1)**  **PERM1** | **Forward Reverse Forward Reverse Forward**  **Reverse** | CCTATCACCCTTGCCATCAT GAGGCTGTTGCTTGTGTGAC ATGGAAAGCCTGCCATCATG TCCTTGTTGTTCAGCATCAC ATAGCTCCATGGCCCTAGCTG  TGGAAGAACCAGGGACAGAC |
| HPRT  TFAM  PGC-1a | **Forward Reverse Forward Reverse**  **Forward** | AGGGATTTGAATCACGTTTG TTTACTGGCAACATCAACAG TTTCCAAGCCTCATTTACAAGC AAACCAAAAAGACCTCGTTCAG  GGTCACTGGAAGATATGGCA |

**Reverse** AGAAGTCCCATACACAACCG
